# Prof. Brainard’s Visit to Europe

**Published:** 1865-12

**Authors:** 


### PROF. BRAINARD'S VISIT TO EUROPE.

Prof. Brainard has left this city, and in the early part of January will sail from New York for Europe, where his family has been some months past. He goes for respite from the heavy professional labors from which he could not escape while here, for improved facilities for scientific investigation, to renew his association with the medical savans of the old world, and to observe what has been done and is now doing in the profession abroad. He will return in the autumn of 1866, in time to give his next course of lectures in the Rush Medical College. When he left, the class of students which he has instructed this winter, marched in procession to the train in which he took his departure, to offer to him their parting salutation, and as a token of their respect, attachment and esteem. Relating to this subject, we have received the following notice of a meeting of the class, the resolutions presented to Prof. Brainard, and his reply to the same:

CHICAGO, Dec. 29th, 1865.

At a meeting of the Students of Rush Medical College, called to consider the departure of Professor Brainard for Europe, the following resolutions were unanimously adopted:

LECTURE ROOM RUSH MEDICAL COLLEGE,
December 26th, 1865.

WHEREAS, Having been informed of the purpose of our honored President and Professor of Surgery—Dr. Brainard—to depart soon for Europe, upon a visit of scientific research, and for the restoration of his health; And whereas, the Students of Rush Medical College feel a lively interest in the welfare and prosperity of their distinguished teacher, therefore be it

*Resolved*, That the members of this class do hereby tender to Prof. Brainard their unfeigned regards, with their best wishes for a safe and prosperous passage to the old world, where, temporarily relieved from the arduous duties of the profession he has so long adorned, he may find such quiet and comfort as will speedily and completely restore his health, partially deranged during his long continued services, faithfully performed, in the advancement of science.

*Resolved*, That while we sincerely regret the necessity of his departure before the expiration of his present collegiate term, we do cheerfully submit to our present loss, believing that the profession at large will be benefited—no little—by his dipping pens, and comparing notes with the medical savans of the east—the Philosophers of the Old World.

*Resolved*, That while we invoke all the pleasures and benefits to be derived from a sojourn with his trans-Atlantic friends and admirers, we do most humbly petition for the Doctor a safe and pleasant "homeward-bound," where, again arrived, he may—by the providence of God—live long to enjoy the fruits of his attainments in that profession which is proud to acknowledge him as one of its bright and leading men.

*Resolved*, That a copy of these proceedings be presented to Professor Brainard, and that copies be furnished THE CHICAGO MEDICAL JOURNAL and city press for publication.

JNO. W. CRAIG,
M. P. SIGWORTH,
N. T. QUALES,
G. M CHAMBERLIN,
C. F. KNAPP,
*Committee on Resolutions.*

Unanimously adopted.

J. B. EGBERT, *President.*

W. W. MURRAY, *Secretary.*

---

CHICAGO, Dec. 27th, 1865.

*To Messrs. Jno. W. Craig, M. P. Sigworth, N. T. Quales, G. M. Chamberlin, C. F. Knapp,*

Committee of the Class of Rush Medical College.

GENTLEMEN:

I beg you to convey to the class my heartfelt thanks for the kind and too flattering resolutions which they adopted and presented to me.

My interest in the institution with which we are all connected, is unabated, and must remain so as long as I live.

It will, therefore, be my pleasure as well as my duty, to resume, at the commencement of the next course of lectures, my position as a teacher in Rush Medical College.

Please convey to the members of the class, gentlemen, my grateful acknowledgments for their kindness, and my warmest wishes for the welfare of each one of them. Accept, also, for yourselves, the thanks and kind wishes with which I shall ever remain,

Yours most truly,

DANIEL BRAINARD,

*Pres. and Prof. of Surgery in Rush Med. College.*

